# Is automated screening for diabetic retinopathy indeed not yet ready as stated by Grauslund et al.?

**DOI:** 10.1111/aos.14236

**Published:** 2019-09-01

**Authors:** Frank D. Verbraak, Ursula Schmidt‐Erfurth, Andrzej Grzybowski, Michael Abramoff, Reinier Schlingemann

**Affiliations:** ^1^ Department of Ophthalmology Amsterdam University Medical Center Amsterdam The Netherlands; ^2^ Department of Ophthalmology Medical University Vienna Vienna Austria; ^3^ Department of Ophthalmology Uniwersytet Warminsko‐Mazurski Olsztyn Poland; ^4^ Department of Ophthalmology and Visual Sciences University of Iowa Hospitals and Clinics Iowa City Iowa USA

Editor,

In a recent paper by Grauslund et al. (2018), the evidence‐based guidelines for screening of diabetic retinopathy (DR) in Denmark were presented. The authors addressed several aspects of screening: classification of DR, examination techniques, screening interval and the possible role of automated screening.

The authors are a group of retinal specialists formed by the Danish Ophthalmological Society, but how the guideline was conceived, and whether as systematic approach was used, is not described in detail in the article. In short, they recommend the use of mydriatic two‐field disc‐ and fovea‐centred images, for grading the International Clinical Retinopathy Disease severity score, supplementary optical coherence tomography (OCT) in case of suspicion of macular oedema, and a flexible individualized screening interval.

However, even though they underscore the need for automated screening in light of the increasing burden of any screening program for DR, with numbers of patients with diabetes mellitus expected to rise considerably in the coming years, they considered automated screening not yet ready for implementation.

The authors acknowledge that the performance in sensitivity (reportedly up to 96%) and specificity (reportedly up to 98.5%) of the latest artificial intelligence‐based algorithms for automated screening is high enough, but they have two arguments against the present use of automated devices for DR screening in Denmark. The first is based on assumed differences between study populations and the real‐world Danish screening population, the second is the assumption that most of these algorithms use single‐field non‐mydriatic fundus images.

Four recent studies, validating different artificial intelligence‐based devices for DR screening have included a wide variety of patients, all representing real‐life populations, with a mix of multiple ethnicities (Gulshan et al. 2016; Ting et al. 2017; Abramoff et al. 2018; Verbraak et al. 2019). These studies showed that the tested devices have a high accuracy with high sensitivity and specificity, against various reference standards. Most importantly, one of these studies was a preregistered clinical trial overseen by FDA, and compared to AI‐based system to a patient outcome proxy and was powered to measure equity, that is comparable accuracy for different races and ethnicities. Based on these studies, the conclusion can be drawn that any population studied appropriately can be reliably screened using these devices, irrespective of the kind of patients screened. Differences in the prevalence of DR, or in the degree of severity of DR encountered in screening populations, can also not be used as an argument against the use of these devices, as the distribution of DR severity in these studies mimics that in real‐world populations (Verbraak et al. 2019).

With the exception of the study by Gulshan et al., the other three studies used fundus photographs as recommended by the authors, including one optic disc‐centred and one fovea‐centred fundus image, and not only a single fovea‐centred image (Gulshan et al. 2016; Ting et al. 2017; Abramoff et al. 2018; Verbraak et al. 2019).

The pivotal trial conducted by Abramoff et al., which lead to FDA approval of the IDx‐DR device was preregistered, and used analysis of the device on these two standard fundus images, and compared this with the only known patient outcome proxy for DR, the 4‐widefield stereo images, covering the seven fields defined in the ETDRS classification studies, graded by the Wisconsin reading centre, combined with OCT‐based diagnosis of centre‐involved diabetic macular oedema as the gold standard (Abramoff et al. 2018). Even against this high level of accuracy of the gold standard, the sensitivity and specificity of this device were very high.

We would like to argue that at present, AI‐based devices for screening of DR are indeed ready to be implemented into the care of our patients with diabetes mellitus. These devices will not only help to reduce the growing burden of screening for DR, but improve the accuracy of screening compared to single human graders (Verbraak et al. 2019).

In line with our conclusion, we have noted that in a more recent article, the first author of the Danish Guideline article, J. Grauslund, appears to agree, stating that ‘Considering both strengths and limitations, as well as the high performance of deep learning‐based algorithms, automated DR classification using deep learning could be feasible in a real‐world screening scenario’ (Nielsen et al. 2019).
